# The complete mitochondrial genome of *Ancherythroculter kurematsui* (Cypriniformes: Cyprinidae)

**DOI:** 10.1080/23802359.2016.1214547

**Published:** 2016-09-05

**Authors:** G J Wang, Z L Zheng, E M Yu, J Xie, N Wei, J R Wu, J S Li

**Affiliations:** a Pearl River Fisheries Research Institute, Chinese Academy of Fishery Science, Guangzhou, Guangdong, China;; b Southwest University Rongchang Campus, Chongqing, China

**Keywords:** *Ancherythroculter kurematsui;* mitochondrial genome, phylogenetic analysis, sequencing

## Abstract

*Ancherythroculter kurematsui* (*A. kurematsui*) is a unique small-size freshwater fish in southwest China. In this study, the complete mitochondrial genome of *A. kurematsui* was determined (GenBank accession number is KU234534). The mitochondrial genome sequence of *A. kurematsui* was a circular molecule with 16,621 bp in length, and contained 37 typical animal mitochondrial genes including 2 ribosomal RNA genes, 13 protein-coding genes, 22 transfer RNA genes and a control region (D-loop). Four nucleotide compositions and their relative proportions of the entire mitogenome was 27.69% C, 16.16% G, 31.21% A and 24.93% T, with an A + T and G + C contents being 56.14% and 43.86%, respectively.

*Ancherythroculter kurematsui* (*A. kurematsui*), a unique small-size freshwater and economic fish species, in southwest China, belongs to the family Cyprinidae and the order Cypriniformes (Liu et al. 2011). It is mainly found only in the upper stream of Yangtze River and its tributary (Ding 1994; Yu 2012). In this study, we first determined the complete mitochondrial genome of *A. kurematsui*, which provides the basic molecular data for the further study on its systematics and conservation biology (Peng et al. 2006).

The complete mitochondrial genome of *A. kurematsui* was found to be a circular molecule with 16,621 bp in length, and has been deposited in GenBank database with accession number of KU234534. The test fish sample was collected from Jialing River in the upper reaches of the Yangtze River, Langzhong City, Sichuan Province, China (31°36′27N″, 106°0′8″E). The specimens were immersed with formaldehyde, and collected in the museum of Pearl River Fisheries Research Institute with accession number SC20151015003.

Its DNA was extracted from the tail muscles using the salting-out procedure (Howe et al. 1997). We completely sequenced the genomic DNA of *A. kurematsui* using high-throughput sequencing technologies on the Illumina HiSeq2500 platforms (San Diego, CA). Twenty-two transfer RNA (tRNA) genes were identified using the tRNAscanSE 1.21 program (Schattner et al. 2005).

As in those of the other vertebrates (Boore 1999), mitochondrial genome of *A. kurematsui* contained 2 ribosomal RNA (rRNA) genes (12S rRNA and 16S rRNA), 13 protein-coding genes, 22 tRNA genes and a control region (D-loop). The overall base compositions of *A. kurematsui* were C = 4603 (27.69%), G = 2686 (16.16%), A = 5188(31.21%) and T = 4144 (24.93%). Except for NADH dehydrogenase subunit 6 (ND6) and eight tRNA genes which were encoded by the light (L)-strand, all the other mitochondrial genes were encoded on the heavy (H)-strand. All the protein-coding genes began with the ATG start codon except for cytochrome c oxidase subunit I (COX1) that started with GTG. The other six protein-coding genes, ND1, COX1, ND4L, ND5, ND6 and ATP synthase subunit 8 (ATP8), harbored the typical termination codon TAA or TAG, whereas the other seven genes, ND2, COX2, ATP6, COX3, ND3, ND4 and cytochrome b (CYTB), had an incomplete termination codon T or TA.

As in the other vertebrates (Inoue et al. 2000), 12S rRNA and 16S rRNA were located between the tRNA^Phe^ and tRNA^Leu^ genes and separated by the tRNA^Val^ gene. The AT contents of the two rRNA genes were 50.67% and 57.19% respectively. Twenty-two tRNA genes were interspersed between rRNA genes and protein-coding gene. D-loop (936 bp) was located between tRNA^Pro^ and tRNA^Phe^, with a high AT content of 63.89%.

The phylogenetic tree was constructed on the basis of the complete mitochondrial genome sequences from *A. kurematsui* and the mitochondrial genome of the other 10 most closely related fish species in the GenBank database by the maximum parsimony method (Figure 1). Numbers on the nodes correspond to bootstrap values based on 1000 iterations.

**Figure 1.**
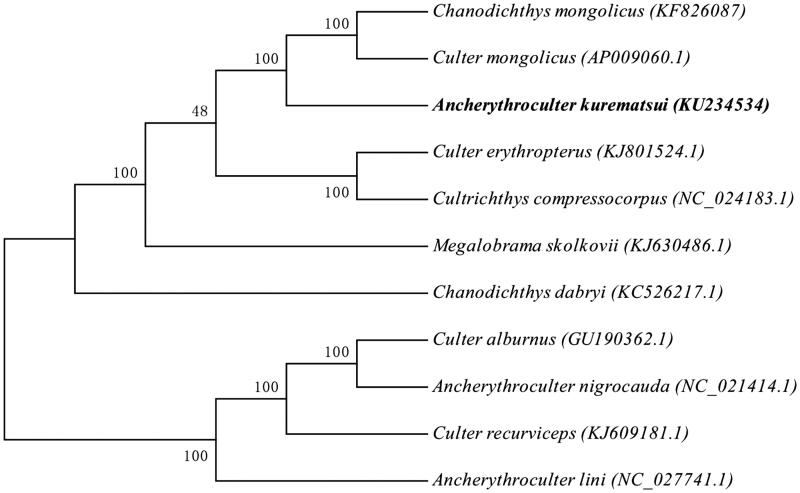
The phylogenetic tree was generated using the RAxML8.1.5. (Pennsylvania State University, PA).
